# Prevalence of Overweight and Obesity among Primary School Students in Split, Croatia

**DOI:** 10.3390/nu16203488

**Published:** 2024-10-15

**Authors:** Ivan Šimunović, Dina Mrčela, Željka Karin, Zenon Pogorelić, Joško Markić

**Affiliations:** 1 School of Medicine, University of Split, Šoltanska 2a, 21000 Split, Croatia; 2 Teaching Institute of Public Health of Split-Dalmatian County, Vukovarska 46, 21000 Split, Croatia; 3 Department of Pediatric Surgery, University Hospital of Split, Spinčićeva 1, 21000 Split, Croatia; 4 Department of Pediatrics, University Hospital of Split, Spinčićeva 1, 21000 Split, Croatia

**Keywords:** body mass index, children, obesity, overweight, health, sports participation, parents’ education

## Abstract

The rising prevalence of obesity among children and adolescents is a global public health concern, significantly contributing to noncommunicable chronic diseases such as cardiovascular disease, diabetes and cancer. In Croatia, obesity rates are alarmingly high, affecting both children and adults. Data from the Institute of the Public Health of Split-Dalmatian County were analyzed to identify trends in body mass index (BMI) among primary school students in Split, Croatia and possible connection with sports participation and parental education levels. A longitudinal analysis of students was conducted across three medical examinations during primary school education. The findings showed an increase in the prevalence of overweight and obesity from 23.4% in the first to 30.2% in the fifth grade, returning back to 23.4% in the eighth grade. Significant differences among students were associated with parental education level. Boys who participated in sports demonstrated a higher BMI in the first grade but there was no difference in BMI in later grades. Sports participation had no significant impact on the BMI of girls. The study underscores the critical role of parental education in influencing children’s weight status and highlights the necessity of tailored public health interventions to address these trends from an early age. The implementation of comprehensive strategies, including educational programs and policy measures as part of the national health policy, is the only way to combat childhood obesity and promote long-term health benefits.

## 1. Introduction

Noncommunicable chronic diseases (NCDs) are long-lasting diseases that progress slowly, are rarely fully curable, and significantly reduce quality of life [1]. Major NCDs include cardiovascular diseases, respiratory diseases, diabetes, and cancers [2]. Initially prevalent only among older adults in industrialized nations, NCDs now affect younger adults globally as well [3,4]. In Croatia, 92% of mortality is attributed to NCDs [5]. Risk factors for the development of NCDs are hypertension, obesity, smoking, excessive alcohol consumption, sedentary lifestyle, unhealthy diet, and excess weight appearing early in life [6]. Adolescents face increased rates of obesity, depression, and reduced quality of life due to physical inactivity and sedentary behaviors [7]. Obesity is defined as excessive fat accumulation that poses health risks and is commonly assessed using body mass index (BMI) [8]. Since 1990, obesity rates have nearly doubled, affecting over 1.9 billion adults and contributing to numerous NCDs. This epidemic also spread to a younger population, affecting millions of children and adolescents [9]. The World Obesity Federation (WOF) emphasizes being overweight as one of the leading risk factors for death, with projections suggesting over four billion individuals will be overweight or obese by 2035 [10]. European countries, including Croatia, face alarming increases in obesity rates among children, adolescents, and adults [11]. Changes in lifestyle and societal factors, such as the availability of unhealthy food and sedentary habits, have accelerated this trend [11,12]. The consequences of childhood and adolescent obesity are extensive, including increased risks of hypertension, type 2 diabetes, cholelithiasis, orthopedic problems, and psychological issues [13,14,15]. Early obesity prevention is crucial to reducing the long-term impact of NCDs [16].

The aim of this study was to analyze trends in BMI, body weight, and body height among primary school students in Split, Croatia and assess a possible connection with sports participation and parental education levels. Furthermore, the study examined the connection between student’s sports participation and their parents’ education.

## 2. Materials and Methods

### 2.1. Participants

The research was conducted through a retrospective analysis of medical records collected by the School and Adolescence Medicine Service of the Public Health Institute of Split-Dalmatia County. Considering that every year approximately 1750 preschool children enroll in primary schools in Split, a sample size of 316 was estimated for confidence level of 95% and 5% margin of error. Using the random selection method, 19 primary schools with 33 class departments were selected to ensure a representative sample. The total number of eligible students in those classes were 560. An inclusion criterion was regular attendance at three systematic examinations. Exclusion criteria were age less than six years at the time of the first examination, insufficient medical data, and children who repeated a grade or delayed enrollment in elementary school due to physical disability or serious chronical health conditions. Also, children who developed serious chronical health condition during primary school education, as well as those on corticosteroid or growth hormone replacement therapy, were excluded.

### 2.2. Data Collection and Description 

The same group of students was followed throughout their primary education, specifically during three examinations. The first mandatory medical examination was held in March 2016, six months before school enrollment (September 2016) and the beginning of primary school education. The following examinations were in the fifth grade (April 2021) and in the eighth grade of primary school (September 2023).

The specialist of school and adolescent medicine collected medical data during examinations. Data were exported from the Complete Prevention software (Cuspis d.o.o., Zagreb, Croatia) and transferred into an Excel spreadsheet for analysis. The collected data included sex (male/female), body weight (BW), body height (BH), grade (1/5/8), sports participation (yes/no), and parents’ education level (primary education, secondary education, and higher education). Anthropometric variables were measured using standardized equipment (Seca Instruments Ltd., Hamburg, Germany) by an experienced technician. The BH was measured to the nearest 1 cm, and BM was reported to the nearest 0.1 kg. To avoid diurnal variation, all the children were tested at the same time of the day (between 7 and 9 a.m.). Body mass index (BMI) was calculated using the following equation: BMI = BM (kg)/BH (m^2^). Weight status was assessed using the World Health Organization BMI reference values [17]. Additionally, to determine the actual participants’ status in BMI levels, we calculated the BMI z-score. This is a measure of how many standard deviations (SD) an individual is above or below the reference data for a specific age and sex since, for children and adolescents, the BMI z-score is age- and sex-specific. Exact ages were not calculated; instead, threshold values for BMI categories were set based on the anticipated average age of children during each examination period: 6.5 years for the first grade, 11.5 years for the fifth grade, and 14 years for the eighth grade.

A z-score of 0 indicates a value that is equal to the mean of the reference group, a positive z-score indicates a value above the mean, and a negative z-score indicates a value below the mean. Weight status categories were determined by deviations in BMI z-scores, expressed as SD from the mean. Defined weight categories were: Overweight >+1 SD, Obesity >+2 SD, Thinness <−2 SD, Severe Thinness <−3 SD [18].

### 2.3. Ethical Aspects

This study followed the Declaration of Helsinki of the World Medical Association subsequent amendments or comparable ethical standards. The Ethics Committee of the Teaching Institute of Public Health of Split-Dalmatia County granted approval for this research (approval number: 2181-103-01-24-03; date of approval: 17 January 2024). Patient consent was waived since the research included only a retrospective analysis of the anonymized medical records.

### 2.4. Statistical Analysis

The collected data were entered into electronic spreadsheets using the Microsoft Office Excel (version 16.74) software and analyzed using the Jeffreys’s Amazing Statistics Program (JASP, version 0.16.3, JASP team, Amsterdam, The Netherlands). Categorical data were expressed as counts or percentages. When comparing categorical data among the observed groups, the chi-square test was used. The distribution of data was tested using the Shapiro–Wilk test. Continuous data that were normally distributed were presented as mean ± SD, while those that were not normally distributed were presented as median with interquartile range (IQR). For normally distributed data, the Student’s *t*-test was used, and for non-normally distributed data, the Mann–Whitney U test was employed.

## 3. Results

### 3.1. Weight Status and Prevalence of Overweight and Obesity

**Figure 1:**
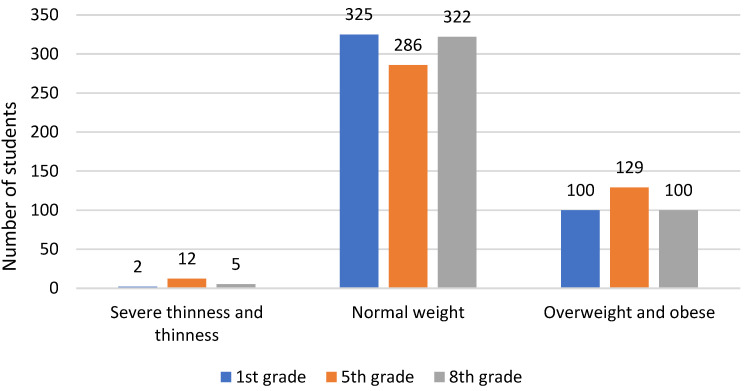
Graphical representation of body mass categories based on the z-score of BMI (*n* = 427).

A total of 560 (32%) out of 1752 students who enrolled in the first grade of primary school in the city of Split in 2016 were included in the study. Out of them, 427 (76.3%) were included in the analysis: 218 girls (51.1%) and 209 boys (48.9%). Weight status categories were created based on deviations in BMI z-scores as defined by World Health Organization standards (Figure 1).

The majority of students across all three systematic examinations were classified as normally nourished but showed a decrease from 76.1% in the first grade to 67% in the fifth grade and an increase to 75.4% in the eighth grade. The prevalence of overweight students slightly increased with age: in the first grade 16.4% students were overweight, rising to 20.4% in the fifth grade, and slightly decreasing to 17.6% in the eighth grade. The number of children with obesity was 7% in the first grade, increased to 9.8% in the fifth grade, and then decreased to 5.9% by the eighth grade.

**Figure 2:**
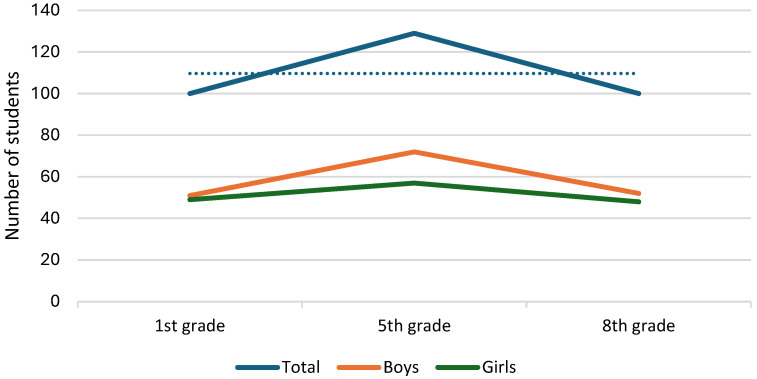
Graphical representation of overweight and obesity prevalence based on sex difference (*n* = 427).

The results indicate a trend of increasing prevalence of overweight and obesity among students at fifth grade examination (Figure 2), with the total number of affected students rising from 100 in the first grade (51 boys, 49 girls) to 129 in the fifth grade (72 boys, 57 girls), before slightly decreasing to 100 in the eighth grade (52 boys, 48 girls). Boys consistently had a higher number of cases compared to girls at each grade level, but the differences between sexes were not statistically significant at any grade level (first grade *p* = 0.639, fifth grade *p* < 0.062, eighth grade *p* < 0.485).

### 3.2. Sex Differences between Anthropometric Indices

Regarding body weight, no significant sex differences were found in the first and fifth grades. However, in the eighth grade, the median body weight of boys was 3 kg higher than that of girls (59.5 kg vs. 56.5 kg; *p* = 0.002). Statistically significant differences in height between boys and girls were observed during all three examinations (*p* < 0.05). No statistically significant differences in BMI z-score were observed during any of the three examinations (*p* > 0.05) (Table 1).

### 3.3. Sports Participation and Differences in Anthropometric Indices

BMI, BMI z-score, body weight, and body height were analyzed between boys and girls regarding their participation in sport (Table 2). In the first grade, boys who participated in sports had significantly higher body weight, BMI, and BMI z-score compared to those who did not participate in sports. In the fifth and eighth grades, there were no significant differences in measured anthropometric indices based on sports participation among boys. Among girls, no significant differences were observed in any of the measured parameters based on participation in sports throughout the study period (Table 2).

### 3.4. Parents’ Education Level and Differences in Anthropometric Indices and Student’s Sport Participation

Furthermore, anthropometric indices and sports participation were analyzed based on the parents’ educational level. During the first-grade examination, no statistically significant differences were observed in anthropometric indices relative to the parents’ education level. In the fifth grade, children of parents with secondary education (SE) showed statistically significant greater value in weight, BMI, and BMI z-score compared to students of parents with higher education (HE) (Table 3). At the eighth-grade examination, statistically significant higher values were found in BMI and BMI z-score in students of parents with SE. Sports participation significantly differed based on the educational level of the parents. Students whose parents had HE were more frequently involved in sports at the time of all examinations (Table 3).

### 3.5. Weight Status of Students Compared with Parents’ Education Level

In a comparison of parents’ education with their children’s weight status following results were found (Table 4). In the first grade, students of parents with SE exhibited a higher prevalence of overweight and obesity compared to students of mothers with HE. The trend continued with a higher prevalence of overweight and obesity among students whose parents had SE compared to those with HE. The majority of students were normally nourished, but the prevalence of normal weight was consistently higher among children of parents with HE.

## 4. Discussion

Throughout the observed period, we found a trend of increasing BMI in children during their primary education. In the first grade, 23.4% of students were overweight or obese, increasing to 30.2% in the fifth grade, and decreasing to 23.4% in the eighth grade. Across all three examinations, there were no significant sex differences in the frequency of overweight and obesity among the student population. The 2017 English Health Survey also noted no sex differences in the prevalence rates. Their research indicated a higher prevalence, with 30% of children aged 2 to 15 years being overweight or obese, and 17% classified as obese [19]. Similarly, an Australian study reported higher prevalence rates than those found in our research [20]. The KiGGS Wave 2 survey (German Health Interview and Examination Survey for Children and Adolescents, 2014–2017) showed similar results to ours, with 15.4% of German children and adolescents being overweight and 5.9% obese, matching our findings regarding the absence of sex differences and the age-related increase in prevalence [21]. Consistent with findings from recent studies, our research indicates a significant increase in the prevalence of overweight and obesity among students during the fifth-grade examination conducted in April 2021, one year after Croatia implemented COVID-19 control measures. The prevalence increased by 6.8%, aligning with global reports indicating weight gain among children and adolescents due to changes in dietary behaviors, increased food intake, and reduced physical activity during the pandemic [22]. CroCOSI 2021/2022 (Childhood Obesity Surveillance Initiative, Croatia) showed a significantly higher prevalence of overweight and obesity among children. Their research indicates that 36.1% of Croatian children aged 8–9 are obese or overweight. In the Adriatic region, the rate of children with overweight and obesity was 38.2%, of which 15.2% were obese [23]. Prevalence was also higher in the rural areas than in cities and among boys. Only one in three overweight children was perceived by their parents as overweight, and obesity was almost entirely overlooked. Therefore, it is crucial to continuously raise awareness and strengthen public health interventions to improve parental health literacy. Considering the previous two rounds of CroCOSI conducted in 2015/2016 and 2018/2019, it is evident that the prevalence of overweight and obesity in children is steadily increasing [23]. The reasons for a such difference between this research and our results are probably due to limitations of our study. The fact that we exclusively analyzed urban population and that the percentage of higher educated parents in our study is higher in the general population in Croatia warrants further research.

The results indicate significant correlations between the prevalence of overweight and obesity in children and the educational level of their parents. Children whose parents have higher educational qualifications show significantly lower rates of obesity compared to those whose parents have secondary education. Throughout the study period, children of mothers with secondary education consistently had a higher prevalence of overweight and obesity. In the first grade, 27.5% of children of mothers with secondary education were overweight or obese, compared to 19.4% of those with higher educated. This trend continued into the fifth and eighth grades with the prevalence rising to 35.5% and 28% for children of mothers with secondary education, compared to 25.5% and 19% for those with higher educated mothers. The same pattern was observed for fathers’ education. In the first grade, 27.9% of children of fathers with secondary education were overweight or obese, compared to 18.2% of children whose fathers were higher educated (*p =* 0.017), and this difference was even more pronounced in the fifth grade, with prevalence rates of 36.2% for children of fathers with secondary education and 23.2% for those with higher educated fathers. These findings indicate a link between lower parental education levels and higher rates of overweight and obesity in children. Supporting our findings, a study by Ruiz et al. across several European countries showed that children of mothers with lower education levels were at higher risk of overweight and obesity, emphasizing the crucial role of maternal education on children’s health outcomes [24]. Similarly, Lamerz et al. found a significant correlation between parental education and childhood obesity, identifying maternal education as a key independent factor for a child having a BMI above the 90th percentile [25]. The observed importance of maternal education in influencing a child’s obesity and overweight can be attributed to mothers traditionally spending more time with their children and playing a more significant role in their nutrition and upbringing [26,27]. Parental decisions about household food consumption are influenced by several factors, including socioeconomic status, education level, cultural heritage, and personal health beliefs. Higher educated parents often have greater nutritional knowledge and awareness, leading to healthier food choices consistent with nutritional guidelines, such as including fruits, vegetables, and whole grains while limiting processed foods and snacks high in fat [28]. Van Ansem et al. found that higher maternal education is associated with healthier eating habits in children, highlighting the importance of educating parents to improve children’s dietary habits [29]. Parents can learn to create a healthy eating environment at home, provide opportunities for physical activity, and discourage sedentary behaviors such as excessive TV watching [30]. Another significant finding from this study is that children of highly educated parents participate more frequently in sports compared to those whose parents have secondary education. In the first grade, 69.9% of children whose mothers had HE were involved in sports, compared to 54.5% of children whose mothers had SE (*p* = 0.002). Similar results were found for fathers, 71.2% of children with HE fathers participated in sports, compared to 55.5% with SE fathers (*p* < 0.001). This trend of significant difference continued at the fifth and eighth grade examination, where children with HE parents more likely participated in sports. This pattern suggests a strong correlation between parental education levels and children’s involvement in sports activities. This phenomenon is confirmed in the existing literature [31,32]. Vermeiren et al. found that children of highly educated mothers have better cardiorespiratory fitness and stronger handgrip strength, with highly educated parents often having higher awareness of the health benefits of physical activity and participating in it themselves [33].

In our study, BMI and body weight were analyzed in boys in the first, fifth, and eighth grades, considering their participation in sports. In the first grade, boys who participated in sports had significantly higher body weight and BMI compared to non-participants. This might be due to the limitation of BMI as a measure of overweight and obesity. BMI has a high specificity for detecting excess body fat, but its sensitivity is limited, making it unreliable at the individual level. A major limitation is the inability of BMI to distinguish between body fat mass and skeletal muscle mass or to assess the distribution of body fat [34,35]. It was noted by Ara et al. in Spain that boys involved in sports showed a greater increase in BMI due to muscle mass increase [36]. In the fifth and eighth grades, there was no significant difference in BMI based on sports participation among boys. No significant differences were observed in girls based on sports participation throughout the study period. This exclusion might be due to variations in the types of sports, intensity of participation, or other socio-cultural factors influencing girls’ physical activities [37,38]. Additionally, factors such as the frequency and consistency of training, different motivational levels, and varying access to sports facilities may further contribute to the lack of observed impact on BMI and BMI z-scores. These factors suggest that not all sports equally influence body composition, highlighting the need for targeted interventions that consider the nature and quality of physical activity rather than merely participation [39,40]. This finding suggests that while sports activities can be beneficial, there might be a need to tailor sports activities to achieve similar health benefits for broader population [41].

Obesity and overweight remain significant global health concerns. Obesity is a major risk factor for NCDs, which are leading causes of diminished quality of life and mortality worldwide. Thus, preventing obesity from an early age during childhood and adolescence is crucial [42]. Effective prevention can be achieved through public health policies that promote healthy eating and physical activity and highlight the dangers of sedentary lifestyles and unhealthy diets [43]. The World Health Organization (WHO) has recognized this issue and introduced guidelines believed to help curb and prevent obesity. These measures include taxes on sugary drinks, bans on advertising unhealthy foods, and improvements to school menus [44].

Based on the study analysis, it is essential to increase parental awareness about healthy eating, physical activity, and the long-term impact of obesity on the development of chronic diseases. By enhancing awareness and educating parents, their lifestyle choices and consequently those of their children can be significantly influenced. Educating parents lays the groundwork for implementing advanced public health policies, such as banning advertisements for sugary drinks and high calorie foods and changing school menus [45]. Our study is the first one to analyze the prevalence of overweight and obesity among primary school students in an urban setting in Croatia. Similarly to the other studies [23], it is evident that the prevalence of overweight and obesity in children is steadily increasing and that a country’s public health authorities need to implement measures to revert this process. One example of governmental action is The Governments of the Republic of Croatia Action Plan for Obesity Prevention 2024–2027, which aims to reduce obesity rates through measures focused on promoting healthy living, preventing risk factors, and strengthening public health and other initiatives aimed at identifying, monitoring, and treating obesity [46]. Implementing comprehensive strategies, such as those outlined in Croatia’s Action Plan for Obesity Prevention 2024–2027, is crucial. These strategies should focus on raising public awareness, encouraging physical activity, and promoting healthy dietary habits from an early age to reduce the long-term health risks associated with childhood obesity.

In addition to the ones previously mentioned, there are several other limitations to this study. First, as it was conducted through a retrospective data analysis, the data collection process could not be controlled. Other variables were not considered that might affect body weight, BMI, and sports participation, such as screen time, dietary habits, or socioeconomic status. During these examinations, the exact ages of students were not considered; all students were treated as the same age regardless of their birth month, despite the WHO categorizing weight status based on monthly BMI z-scores.

## 5. Conclusions

This study highlights the increasing prevalence of overweight and obesity among primary school students in Split, particularly noting a significant correlation between higher BMI and lower parental education levels. Students of parents with lower educational attainment were more likely to be overweight or obese, with this disparity becoming more pronounced in higher grades. Notably, children of highly educated parents were more frequently involved in sports, which had varying impacts on BMI. Boys who participated in sports demonstrated significantly lower BMI in the fifth and eighth grades. On the other hand, sports participation did not significantly affect the BMI of girls. These findings underscore the critical influence of parental education on children’s health outcomes and suggest that tailored public health interventions and educational programs could effectively address these issues.

## Figures and Tables

**Table 1:** Distribution of participants by sex.

	Total *n* = 427	Boys *n* = 209	Girls *n* = 218	*p*
1st grade				
Weight (kg)	25 (22, 28)	25.5 (23, 28)	24 (22, 27.5)	0.085 *
Height (m)	1.26 ± 0.06	1.26 ± 0.06	1.25 ± 0.06	0.039 ^⸸^
BMI (kg/m^2^)	15.80 (14.69, 19.91)	15.80 (14.78, 16.83)	15.80 (14.65, 17)	0.765 *
BMI z-score	0.30 ± 1.09	0.33 ± 1.12	0.28 ± 1.07	0.67 ^⸸^
5th grade				
Weight (kg)	45 (39, 52.5)	45 (38, 52)	45 (40, 52.5)	0.293 *
Height (m)	1.56 ± 0.08	1.55 ± 0.08	1.56 ± 0.08	0.019 ^⸸^
BMI (kg/m^2^)	18.38 (16.73, 21.18)	18.46 (16.78, 21.23)	18.32 (16.73, 21.14)	0.871 *
BMI z-score	0.29 (−0.52, 1.26)	0.43 (−0.43, 1.01)	0.13 (−0.59, 1.01)	0.052 *
8th grade				
Weight (kg)	58 (52, 65.75)	59.5 (53, 69)	56.5 (51.5, 63)	0.002 *
Height (m)	1.71 ± 0.08	1.74 ± 0.08	1.68 ± 0.06	0.001 ^⸸^
BMI (kg/m^2^)	19.77 (18.07, 22.14)	19.49 (17.9, 21.72)	19.95 (18.36, 22.24)	0.165 *
BMI z-score	0.15 (−0.51, 0.93)	0.20 (−0.52, 0.98)	0.14 (−0.48, 0.87)	0.522 *

*—Mann–Whitney U test; ⸸—Student’s *t*-test; *p* < 0.05 (statistically significant); BMI—body mass index; BMI z-score indicates individual’s BMI value relative to reference population; values are presented as mean (±SD) or median (IQR).

**Table 2:** Distribution of participants by sex and sports participation.

	Sports Participation—Girls (*n* = 218)			Sports Participation—Boys (*n* = 209)		
	Yes	No	*p*	Yes	No	*p*
1st grade	*n* = 131	*n* = 87		*n* = 137	*n* = 72	
Weight (kg)	24 (22, 27)	24 (22, 28)	0.641 *	26 (23, 28)	26 (21.5, 26.63)	0.017 *
Height (m)	1.25 ± 0.05	1.25 ± 0.06	0.704 ^⸸^	1.26 ± 0.05	1.26 ± 0.06	0.127 ^⸸^
BMI (kg/m^2^)	15.8 (14.69, 16.77)	15.81 (14.54, 17.31)	0.538 *	16 (14.96, 16.92)	15.42 (14.55, 16.55)	0.022 *
BMI z-score	0.285 ± 0.97	0.314 ± 1.21	0.641 *	0.437 ± 1.09	0.113 ± 1.14	0.023 *
5th grade	*n* = 144	*n* = 74		*n* = 153	*n* = 56	
Weight (kg)	44.75 (40, 51.63)	45.75 (41.25, 54.5)	0.871 *	44 (38.5, 51)	46 (38, 57.63)	0.820 *
Height (m)	1.56 ± 0.08	1.57 ± 0.07	0.869 ^⸸^	1.55 ± 0.08	1.55 ± 0.08	0.444 ^⸸^
BMI (kg/m^2^)	18.16 (16.66, 21.20)	18.75 (16.98, 20.89)	0.779 *	18.38 (16.88, 20.72)	18.67 (16.74, 22.93)	0.842 *
BMI z-score	0.18 ± 1.17	0.32 ± 1.20	0.789 ^⸸^	0.39 (−0.34, 1.25)	0.51 (−0.42, 1.85)	0.842 *
8th grade	n = 142	*n* = 76		*n* = 149	*n* = 60	
Weight (kg)	55.5 (51.63, 62)	57.25 (51, 67.13)	0.883 *	60 (54, 68)	58.25 (49.75, 72)	0.266 *
Height (m)	1.68 ± 0.06	1.68 ± 0.0	0.635 ^⸸^	1.74 ± 0.08	1.74 ± 0.08	0.275 *
BMI (kg/m^2^)	19.67 (18.40, 21.57)	20.62 (18.15, 23.77)	0.914 *	19.66 (18, 21.53)	19.05 (17.15, 23.77)	0.247 *
BMI z-score	0.39 (−0.46, 0.67)	0.37 (−0.57, 1.26)	0.914 *	0.29 ± 1.01	0.18 ± 1.42	0.281 ^⸸^

*—Mann–Whitney U test; ⸸—Student’s *t*-test; *p* < 0.05 (statistically significant); BMI—body mass index; BMI z-score—individual’s BMI value relative to reference population; values are presented as mean (±SD).

**Table 3:** Distribution of participants based on parental education level (*n* = 427).

	Mother’s Education			Father’s Education		
	SE *n* = 211	HE *n* = 216	*p*	SE *n* = 229	HE *n* = 198	*p*
1st grade						
Weight (kg)	25 (22, 28)	25 (22, 27.5)	0.543 *	25 (23, 28)	24.75 (22, 27)	0.207 *
Height (m)	1.26 ± 0.06	1.26 ± 0.05	0.926 ^⸸^	1.26 ± 0.05	1.25 ± 0.06	0.236 ^⸸^
BMI (kg/m^2^)	15.84 (14.71, 17.2)	15.79 (14.68, 16.70)	0.211 *	15.98 (14.71, 17.22)	15.68 (14.68, 16.66)	0.182 *
BMI z-score	0.37 ± 1.153	0.237 ± 1.026	0.206 ^⸸^	0.4 (−0.43, 1.08)	0.21 (−0.48, 0.83)	0.172 *
Sports participation	117 (54.5%)	151 (69.9%)	0.002 ⁑	127 (55.5%)	141 (71.2%)	<0.001 ⁑
5th grade						
Weight (kg)	46 (40, 55.5)	44 (38, 51)	0.008 *	46 (40, 55.5)	43 (38, 51)	0.002 *
Height (m)	1.56 ± 0.08	1.55 ± 0.08	0.838 ^⸸^	1.56 ± 0.07	1.55 ± 0.08	0.321 ^⸸^
BMI (kg/m^2^)	18.92 (17.24, 21.97)	18.03 (16.35, 20.45)	0.001 *	18.94 (17.19, 21.9)	17.99 (16.37, 20.28)	<0.001 *
BMI z-score	0.12 (−0.28, 1.49)	0.12 (−0.75, 0.98)	0.002 *	0.55 (−0.32, 1.48)	0.1 (−0.74, 0.95)	<0.001 *
Sports participation	134 (63.5%)	163 (75.5%)	0.007 ⁑	149 (65.1%)	148 (74.8%)	0.03 ⁑
8th grade						
Weight (kg)	59 (53, 68)	57 (52, 63.5)	0.017 *	59 (53, 67.5)	57.5 (52, 63.5)	0.467 *
Height (m)	1.71 ± 0.07	1.71 ± 0.08	0.277 ^⸸^	1.71 ± 0.08	1.71 ± 0.08	0.579 ^⸸^
BMI (kg/m^2^)	20.15 (18.4, 23.23)	19.26 (17.92, 21.32)	0.003 *	20.08 (18.31, 22.76)	19.2 (17.9, 21.37)	0.012 *
BMI z-score	0.39 (−0.40, 1.23)	−0.002 (−0.60, 0.75)	0.003 *	−0.006 (−0.43, 1.06)	−0.006 (−0.60, 0.77)	0.012 *
Sports participation	128 (60.7%)	163 (75.5%)	0.001 ⁑	146 (63.8%)	145 (73.2%)	0.036 ⁑

*—Mann–Whitney U test, ^⸸^—Student’s *t*-test, ⁑—Chi-square test, *p* < 0.05 (statistically significant); values are presented as mean (AS) ± standard deviation (SD) or median (Md) and interquartile range (IQR); sports participation (*n*, %); BMI—body mass index; BMI z-score—individual’s BMI value relative to reference population; HE—higher education; SE—secondary education.

**Table 4:** Distribution of participants according to parental education level and weight status.

	Mother’s Education			Father’s Education		
	SE *n* = 211	HE *n* = 216	*p ⁑*	SE *n* = 229	HE *n* = 198	*p ⁑*
1st grade						
Underweight and Normal Weight	153 (72.5%)	174 (80.6%)	0.049	165 (72.1%)	162 (81.8%)	0.017
Overweight and Obese	58 (27.5%)	42 (19.4%)		64 (27.9%)	36 (18.2%)	
5th grade						
Underweight and Normal Weight	136 (64.5%)	162 (75%)	0.018	146 (63.8%)	152 (76.8%)	0.003
Overweight and Obese	75 (35.5%)	54 (25%)		83 (36.2%)	46 (23.2%)	
8th grade						
Underweight and Normal Weight	152 (72%)	175 (81%)	0.028	168 (73.4%)	159 (80.3%)	0.091
Overweight and Obese	59 (28%)	41 (19%)		61 (26.6%)	39 (19.7%)	

Data are presented as frequency (%); ⁑—Chi-square test, *p* < 0.05 (statistically significant); weight status is determined by z-score; categories of weight status based on deviation of the body mass index (BMI) z-score in standard deviations (SD) are: underweight and normal weight < 1 SD, overweight and obese > 1 SD; HE—higher education; SE—secondary education.

## Data Availability

The data presented in this study are available on request from the corresponding author.
